# Extensive layer clouds in the global electric circuit: their effects on vertical charge distribution and storage

**DOI:** 10.1098/rspa.2019.0758

**Published:** 2020-06-03

**Authors:** R. Giles Harrison, Keri A. Nicoll, Evgeny Mareev, Nikolay Slyunyaev, Michael J. Rycroft

**Affiliations:** 1 Department of Meteorology, University of Reading, Reading RG6 6BB, UK; 2 Department of Electronic and Electrical Engineering, University of Bath, Bath BA2 7AY, UK; 3 Institute of Applied Physics, Russian Academy of Sciences, Nizhny Novgorod 603950, Russia; 4 CAESAR Consultancy, 35 Millington Road, Cambridge CB3 9HW, UK

**Keywords:** atmospheric electricity, Earth system, global circuit, clouds, cosmic rays

## Abstract

A fair-weather electric field has been observed near the Earth's surface for over two centuries. The field is sustained by charge generation in distant disturbed weather regions, through current flow in the global electric circuit. Conventionally, the fair-weather part of the global circuit has disregarded clouds, but extensive layer clouds, important to climate, are widespread globally. Such clouds are not electrically inert, becoming charged at their upper and lower horizontal boundaries from vertical current flow, in a new electrical regime—neither fair nor disturbed weather; hence it is described here as *semi-fair weather*. Calculations and measurements show the upper cloud boundary charge is usually positive, the cloud interior positive and the lower cloud boundary negative, with the upper charge density larger, but of the same magnitude (∼nC m^−2^) as cloud base. Globally, the total positive charge stored by layer clouds is approximately 10^5 ^C, which, combined with the positive charge in the atmospheric column above the cloud up to the ionosphere, balances the total negative surface charge of the fair-weather regions. Extensive layer clouds are, therefore, an intrinsic aspect of the global circuit, and the resulting natural charging of their cloud droplets is a fundamental atmospheric feature.

## 1. Introduction

Some of the earliest measurements in atmospheric electricity established the existence of electrification in the atmosphere in fair weather [1–3], subsequently quantified by Kelvin [4] as a downward-directed vertical electric field. Establishing the origin of this field motivated many of the original researchers in the subject of atmospheric electricity. Wilson resolved this problem by suggesting electrical current flow between disturbed and fair-weather regions [5,6] and providing a conceptual explanatory framework now known as the global electric circuit (GEC) [6].^1^ The downward fair-weather electric field lines imply that the Earth carries a negative charge density on its land and ocean surface. For the atmospheric system to be neutral overall, a positive charge is, therefore, required in the fair-weather atmosphere. The distribution of fair-weather atmospheric positive charge is discussed further here in the context of the complicating effects of layer clouds, which have conventionally been neglected in many studies of the GEC.

Throughout the natural atmosphere, horizontal layer structures are common. For example, up to about 3 km from the surface, there is a planetary boundary layer (PBL) within which drag from the roughness of the surface has its greatest effects, and above which, in the so-called free troposphere, the atmospheric flow is relatively unaffected. This near-surface region can be readily identified in vertical profiles of many properties, such as in balloon ascents of temperature, which show a distinctive step in their values at the top of the boundary layer. Clouds also frequently show a well-defined layer structure, arising from the combined effects of a lower boundary caused by rising moist air cooling to water vapour saturation, and an upper temperature inversion limiting further ascent.

Charge is released in the atmosphere by ionization processes associated with galactic cosmic rays and radioactivity near the Earth's continental surface. Ionization creates positive and negative molecular cluster ions (typically sub-nanometre and conventionally known as *small ions*), which are the principal charge carriers providing the electrical conductivity of air [7]. When aerosol particles or droplets are present, the small ions become attached to the aerosol or droplets, transferring their charge to the larger particles or droplets. The net charge occurring in the lower atmosphere is, therefore, mostly present on a combination of small ions, charged aerosol or charged droplets; their total charge per unit volume is known as the *space charge*. The main regions of space charge within the lowest few kilometres of the atmosphere are within the PBL and near and inside cloud layers. If this space charge is actively transported, e.g. by turbulent processes in the PBL, the net effect can be that currents are generated which can contribute to the global circuit [8].

The typical space charge within the PBL ranges from around 1 pC m^−3^ [9,10] to 100 pC m^−3^ [11], but this depends on the local aerosol properties and vertical distribution as well as the turbulent state of the lower atmosphere; therefore, there is a variation in space charge with local time of day. For example, the existence of an electrode layer which can form in calm conditions, typically nocturnally, immediately adjacent to the surface, has been shown to contain substantial space charge of several hundred pC m^−3^ [12], which disperses by mixing soon after sunrise. Although the vertical profile of aerosol within the PBL influences the conductivity profile, and is, therefore, key to modelling the GEC, the complexity and variability of processes occurring within the PBL mean that the full treatment of this problem is complex and extensive, for example requiring full large-eddy simulations [13,14]. This complexity has to be considered when representing the turbulent convection currents generated within the PBL, which may also contribute to charging currents within the GEC [8]. In contrast to the highly complex and variable nature of charging processes within the PBL, the acquisition of space charge within stratiform layer clouds occurs in an ordered way, because of the flow of small ions comprising the vertical conduction current through the cloud boundary.

A further important aspect of the attachment of small ions to aerosol and droplets is the associated reduction of the electrical conductivity, as the removal of small ions removes mobile charge carriers; the larger aerosol and droplets to which the small ions' charge is transferred are considerably less mobile, and so the conductivity is reduced. This means that, at a boundary between droplets and clear air, the conductivity is less in the droplet-laden region than in the clear air region, forming an electrical transition. (The importance of this electrical transition region was originally recognized by Gunn [15].) Such a situation arises at the upper and lower horizontal edges of layer clouds, or the upper edge of a fog layer. In the natural atmosphere, the global circuit is expected to drive a vertical current through extensive layer clouds and fogs. This has been observed [16,17]; it leads to space charge accumulation at the cloud–air conductivity transition [18,19]. For persistent extensive layer clouds, observations confirm positive space charge accumulation at the upper horizontal transition from the clear air above the cloud, and negative space charge at the lower transition from cloud to clearer air beneath [20]. An extensive layer cloud, therefore, has two electrical effects: firstly, to modify the vertical conductivity profile from that of clear air conditions (which typically increases exponentially with height) [21] and, secondly, to provide boundaries at which accumulation of charge occurs. Hence, as a result of the formation of a persistent layer cloud in which there is negligible internal mixing, an ordered vertical separation of positive and negative charges occurs.

The clouds conventionally considered as ‘generators’ of current within the global circuit are thunderstorms and electrified shower clouds (ESCs) [5], owing to the active charge separation within them. By contrast, clouds in fair-weather regions provide a resistive load in the return part of the circuit [22,23], with layer clouds acting as passive accumulators of charge [19]. Although a full consideration of the effects of the lower atmosphere on the GEC and ionospheric potential requires the inclusion of the electrical processes within the PBL, this is a complex task (for the reasons mentioned previously), beyond the scope of the present paper. In this paper, we focus on the circumstances well away from thunderstorms where there are extensive and persistent layer clouds, which we refer to as semi-fair-weather conditions (defined as situations which can include clouds, but where no substantial charging current is generated). We extend the global atmospheric electric circuit concept to include the role of layer clouds, conventionally overlooked when a solely electrical perspective is taken. Such clouds are known to cover around 30% of the planet at any one time [24].

The structure of this paper is as follows: §2 describes various conceptual approaches to represent the charge distribution in the GEC. In §3, a detailed analysis of the charge distribution in the fair-weather region with layer clouds is presented and illustrated by the results of field observations. Section 4 employs a current-network approach to analyse this charge structure from a theoretical viewpoint, and §§5 and 6 provide further discussion and conclusions, respectively.

## 2. On the distribution of the electric charge in the global circuit

Historically, an analogy between the spherical Earth and a spherical capacitor has provided a useful conceptual geometry with which to represent the DC global circuit [25,26]. It is based on two well-established observational findings:

1. the presence of a fair-weather electric field, directed downwards at the Earth's surface,
2. the presence of downward vertical current flow in fair-weather regions.

Two associated deductions can be made from findings (1) and (2), which are, respectively, that

(A) a negative charge is distributed across the fair-weather part of the terrestrial sphere,
(B) a sustained upper positive potential exists.

Some calculations readily follow from these. Consider deduction (A) first. From finding (1) of the existence of a fair-weather electric field *E*_s_ at the surface, the fair-weather surface charge density *s* can be found from Gauss' law as (hereafter we use SI units)

2.1 $$s=\varepsilon_{0}E_{\text{s}}.$$

Inserting a typical measured fair-weather field (*E*_s_ = −120 V m^−1^) [27] and the permittivity of free space *ϵ*_0_ = 8.85 × 10^−12^  F m^−1^, this gives *s* = −1 nC m^−2^. Assuming that fair-weather regions cover all the Earth (i.e. neglecting disturbed weather regions which occur over only a small portion of the Earth's surface), multiplying *s* by the surface area of the Earth implies that the total charge *Q*_fw_ on the fair-weather part of the planet's surface is as follows:

2.2 $$Q_{\text{fw}}=4\pi R_{\text{E}}^{2}\varepsilon_{0}E_{\text{s}}.$$

Using the value above for *E*_s_ and *R*_E_ = 6370 km, equation (2.2) gives *Q*_fw_ = −5 × 10^5^ C.

This estimate of *Q*_fw_ has been known for a long time; it was thought to represent the total electric charge of the Earth, which was counterbalanced by the charge of the atmosphere [28, ch. 3]. However, it is important to emphasize that such an interpretation is inadequate: *Q*_fw_ is not the total charge on the Earth's surface but only the charge of its fair-weather part. In fact, although thunderstorm regions occupy a much smaller area than fair-weather regions, they are characterized by much greater electric fields, allowing the magnitude of the total positive surface charge beneath them to be of the same order as –*Q*_fw_ [29]. In this study, however, we consider only the fair-weather and semi-fair-weather (i.e. regions with layer clouds) parts of the global circuit.

Let us now consider deduction (B). Because there is a negative (i.e. downward) electric field in fair weather, if the Earth is regarded to be at zero potential, the potential at positions away from the surface must become increasingly positive with altitude. Observations such as those considered by Markson [10] indicate that the rate of increase of fair-weather potential with height (i.e. the potential gradient (PG), representing the electric field magnitude) is not constant; above the boundary layer and outside clouds it decreases exponentially with height and the maximum asymptotic value of potential is reached fairly rapidly, by about 10 km altitude. This potential is the upper potential, *V*_u_; the region in which it occurs was once termed the *electrosphere*. In theoretical studies of the GEC, *V*_u_ is often termed the ‘ionospheric potential’, and a typical value for it is about 240 kV [30]. Without this upper potential the global circuit would not exist. The upper potential is also closely linked to the electrically active solar wind, which affects the magnetosphere, but this is not considered further here.

The existence of the upper potential *V*_u_ together with the negative charge on the fair-weather part of the Earth's surface makes it natural to suppose that the entire GEC can be well approximated by a spherical capacitor with the Earth's surface as the inner electrode and a compensating positive charge on the ionosphere serving as the outer electrode. However, such a representation of the GEC can readily be seen to be misleading and inadequate: the fair-weather electric field magnitude decreases exponentially with altitude and, as mentioned, the potential nearly attains its asymptotic value *V*_u_ at about 10 km (much lower than the height of the ionosphere, approx. 70 km). It, therefore, follows directly from Gauss' law that a substantial amount of positive charge is distributed throughout the lower part of the fair-weather atmosphere: this contradicts the idea of the Earth–ionosphere spherical capacitor. Recently, Haldoupis *et al*. [31] tried to revise the spherical capacitor approach by considering the distributed outer electrode as a compensating positive charge in the lower atmosphere. However, modifying the properties of spherical capacitor models does not alter the fact that they are not the most natural way to represent the DC global circuit. This is because such models are primarily electrostatic, and the equations of electrostatics are only formulated in terms of electric charges, neglecting the fundamentally important aspect that the atmosphere itself is conductive.

The DC global circuit is a system of electric fields and currents maintained by certain source currents (e.g. from charge separation in electrified clouds) in a medium with variable non-zero conductivity. The structure and behaviour of these fields and currents are in many aspects substantially different from those implied by simple electrostatic capacitor models. The most natural way to discuss the global circuit is to represent it as a distributed current network rather than a capacitor; this approach to analysing and modelling the global electrical system has been used since at least the 1950s [32–36]. More recent models of the DC global circuit are also based on this representation; they solve equations for the electric potential in a distributed conductive medium [37–40].

As noted, the lower fair-weather atmosphere contains a large amount of positive electric charge. Given that extensive layer clouds of liquid water (i.e. stratiform clouds) occur in the lower troposphere (below 3 km) where the conductivity is relatively small, and such clouds are abundant, covering about 30% of the planetary surface [24], it is clear that they must also significantly affect the fair-weather charge distribution. A careful and thorough analysis of the charge distribution in the *entire* DC global circuit must be based on considering the semi-fair-weather part of the Earth's surface together with the regions occupied by electrified clouds (which serve as GEC generators). Here, however, the attention is focused only on the electrical structure of the fair-weather part of the DC global circuit in the presence of layer clouds, in which, as far as possible, complications from the direct effects of the global circuit's current generators are neglected. Section 3 presents the electric charge structure in such regions inferred from the results of field observations, while in §4 we shall employ a current-network approach to analyse this charge structure from a theoretical viewpoint.

## 3. Observations of layer cloud charge

### (a). *In situ* measurements

**Figure 1.**
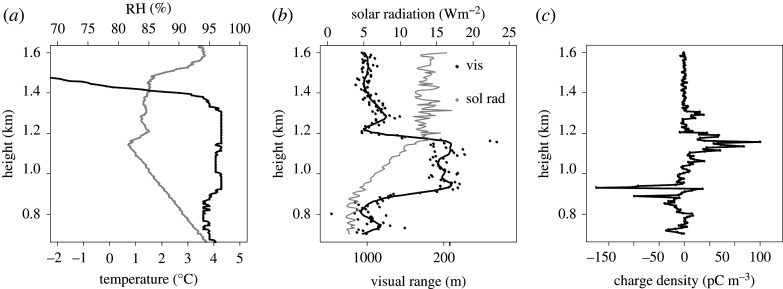
Vertical profile through a stratocumulus layer over Reading University Atmospheric Observatory, UK, from a specially instrumented radiosonde. (*a*) Temperature (grey) and relative humidity (RH) (black) measured by the radiosonde; (*b*) visual range and downwards solar radiation measured by an optical cloud-droplet sensor showing the location of the cloud layer; (*c*) space charge density measured by an electrostatic charge sensor. Adapted from Nicoll & Harrison [20].

**Figure 2.**
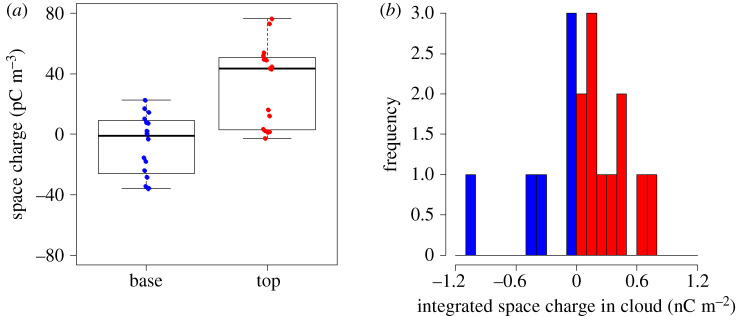
Summary of space charge densities measured from balloon-borne charge sensors at cloud top and cloud base in 17 stratiform clouds above Reading, UK. (*a*) Boxplot of mean charge (thick line represents median) at cloud base (blue) and top (red). Data are averages over cloud top/base regions, where each point represents one cloud edge, for cloud layers with an altitude less than 3 km. Adapted from Nicoll & Harrison [20]. (*b*) Histogram of integrated space charge inside the same cloud layers as in (*a*). The median of the distribution is 0.087 nC m^−2^. (Online version in colour.)

The cloud edge charge density at the bottom and the top can be measured using *in situ* instruments. Nicoll & Harrison [20] reported a series of measurements made using balloon-carried electrometers attached to a meteorological radiosonde. An example flight through a stratiform cloud over the UK is shown in figure 1. This shows well-defined layers of positive charge at cloud top and negative charge at cloud base, with magnitudes reaching approximately 200 pC m^−3^. By sampling many stratiform cloud layers, Nicoll & Harrison [20] also demonstrated that, on average, the upper cloud edge charge density has a greater magnitude than the cloud base charge density. This is because the transition distances from clear air to cloudy air are different at cloud top and cloud bottom, as different physical processes are acting. At the cloud top, the boundary is established by a temperature inversion, and therefore tends to be quite sharp (i.e. the cloud to air transition occurs in a relatively short vertical distance). At cloud base, the effect of updrafts and variability in the condensation level lead to a less well-defined transition. The consequence is that the cloud top boundary is sharper, with a greater gradient in vertical conductivity. As a result, the cloud top charge density is greater, which leads to the cloud carrying a net positive charge. Figure 2*a* shows a boxplot of the space charge densities at cloud top and cloud base from 17 stratiform clouds over Reading, UK. Stratiform cloud bases are typically negatively charged (median charge density by volume −1 pC m^−3^), and the stratiform cloud tops positively charged (median charge density 43 pC m^−3^). The larger magnitude of the charge density at cloud top is also evident. The few anomalous positive cloud base cases may result from turbulence and downward mixing of upper cloud charge, as recently reported by Harrison *et al*. [41].

Figure 2*b* shows a histogram of the integrated space charge within each of the 17 cloud layers (where cloud top and base are defined as where the conductivity gradient starts to change). This demonstrates that, although the clouds typically contain both polarities, on average they have a net positive charge (mean charge density by area 0.087 nC m^−2^).

### (b). Remote sensing cloud base charge measurements

**Figure 3.**
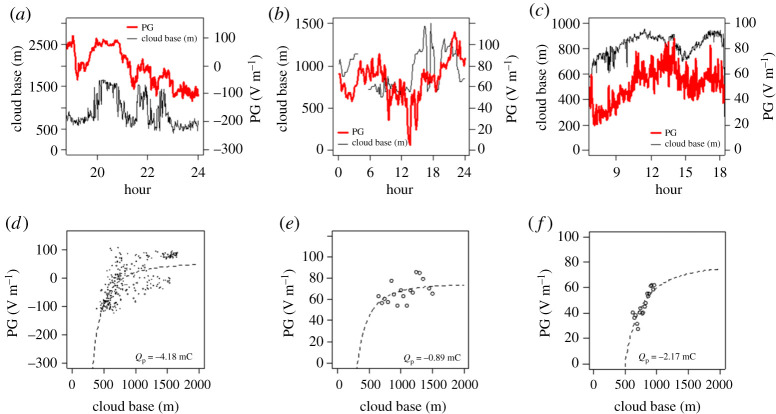
Time series of variations in cloud base height (thin black line) and surface potential gradient (PG) (thick red line), for (*a*) Reading, UK, on 28 February 2018, (*b*) Sodankylä, Finland, on 4 August 2017 and (*c*) Halley, Antarctica, on 20 February 2015. Plots (*d*–*f*) show the mean PG plotted against the cloud base height for the same days at Reading, Sodankylä and Halley, respectively, after binning the cloud base values into 50 m (Sodankylä) and 20 m steps (Halley) (Reading cloud data are at the original 9 m vertical resolution). The equivalent point charge (*Q*_p_) at the cloud base height has been derived in each case, assuming that the cloud base charge remains constant as the cloud base height varies. (Data from Reading and Halley are at 1 min sampling, and from Sodankylä at 10 min sampling.) (Online version in colour.)

Although *in situ* balloon measurements provide a useful instantaneous measurement of layer cloud charge, they can only be used opportunistically, which limits the data available, particularly for assessing the variability of layer cloud charge. A method of remotely sensing cloud charge has, therefore, been developed, based on the principle that low-level charged cloud bases affect the electric field at the surface electrostatically. Recent measurements at Reading show that the surface electric field is influenced for clouds with bases below about 1000 m [42]. As the edge charging process occurs on a more rapid time scale than that of the dissipation of the cloud, if there are slow variations in the cloud base height, the effects of such variations can also appear in surface electric field measurements. This covariation allows the cloud base charge to be inferred and, in some cases when conditions permit, the charge density can be calculated. Figure 3 shows an example of the variation in surface electric field (represented here as PG) with local time and with cloud base height for extensive layer cloud conditions, for three sites widely distributed across the Earth—(*a*) Reading, UK (mid-latitude), (*b*) Sodankylä, Finland (northern polar latitude) and (*c*) Halley, Antarctica (southern polar latitude). At Reading, the relationship between cloud base height and PG is very apparent. For the purpose of identifying and comparing charges, the simplest case of an equivalent representative point charge in the cloud base is considered. This is derived according to the method presented in Harrison *et al*. [42] using a combination of surface PG and cloud base height data from a laser ceilometer, and found to be −4.2 mC for Reading, −0.9 mC for the Sodankylä cloud and −2.2 mC for Halley. These observations demonstrate that negative charging in the base of layer clouds, which is expected to occur globally from theory [18], is indeed a widespread phenomenon.

**Figure 4.**
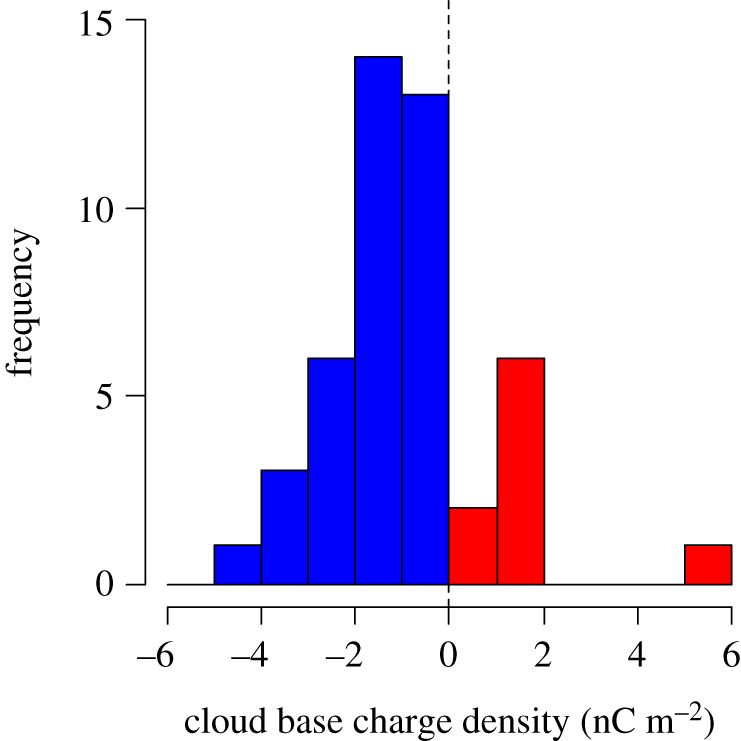
Daily cloud base column charge density, from 2015 to November 2018 (46 valid days), derived from PG and laser ceilometer measurements at Reading, UK, using the methodology discussed in Harrison *et al*. [42] (as per their fig. 4). Negatively charged cloud bases are marked in blue (37 cases), and positively charged cloud bases (nine cases) in red. (Online version in colour.)

Figure 4 shows a summary histogram of daily layer cloud base charge derived from PG and cloud base (ceilometer) measurements between 2015 and 2018 made at Reading, UK, using the remote sensing method detailed in Harrison *et al*. [42]. As for the clouds sampled by the *in situ* method (figure 1), the polarity of the cloud base charge is mostly negative, with median surface charge density −1 nC m^−2^. The occasional occurrence of layer clouds with positive lower charge is likely to be due to the greater effects of dynamical mixing within some clouds (from turbulent and convective processes), which re-distributes charge from the cloud edges. While this method does not determine the cloud top charge, it is clear from the *in situ* measurements of figure 1*a* that both cloud base and cloud top charges are of the same order of magnitude.

These observations lead to the conclusion that stratiform clouds readily acquire charge and, as they are abundant globally, their charge accumulation is likely to form an intrinsic aspect of the global circuit.

## 4. Theoretical aspects of layer cloud charging

The previous section demonstrated the widespread presence of charge in stratiform layer clouds, and the typical magnitude of the charges. In order to determine how such charge influences the vertical distribution of charge within the GEC, a more theoretical approach is required, which is now developed to analyse the entire charge distribution in the atmospheric column containing the cloud layer. The electric potential *ϕ* in fair-weather conditions satisfies the equation

4.1 div(σ grad ϕ)=0,

where *σ* is the conductivity. In the one-dimensional approximation, this becomes

4.2 $$\frac{\text{d}}{\text{d}z}\left(\sigma (z)\frac{\text{d}\phi }{\text{d}z}(z)\right)=0.$$

From this, it follows that the conduction current density, *j_z_*, given by Ohm's law as

4.3 $$j_{z}=-\sigma (z)\frac{\text{d}\phi }{\text{d}z}(z),$$

does not vary with height *z*. The electric field profile is given by

4.4 $$E_{z}(z)=-\frac{\text{d}\phi }{\text{d}z}(z)=\frac{j_{z}}{\sigma (z)}.$$

Hence the charge density, *ρ*, where

4.5 $$\rho =\varepsilon_{0}\mathrm{div}\;\text{E},$$

is expressed by the formula

4.6 $$\rho (z)=\varepsilon_{0}\frac{\text{d}E_{z}}{\text{d}z}(z)=\varepsilon_{0}j_{z}\frac{\text{d}}{\text{d}z}\left(\frac{1}{\sigma }\right)(z)=-\frac{\varepsilon_{0}j_{z}}{\sigma {\left(z\right)}^{2}}\frac{\text{d}\sigma }{\text{d}z}(z)$$

(cf. [18]).

Demanding that *ϕ*(0) = 0, we can infer from (4.4) the potential profile

4.7 $$\phi (z)=-j_{z}\int_{0}^{z}\frac{\text{d}z}{\sigma (z)}$$

and the value of the ionospheric potential (upper potential)

4.8 $$V_{\text{u}}=-j_{z}\int_{0}^{h_{\text{u}}}\frac{\text{d}z}{\sigma (z)},$$

where *h*_u_ is the height of the upper boundary of the model atmosphere. In GEC models this height *h*_u_ is usually set at the lower limit of the ionosphere (where the conductivity becomes essentially anisotropic) and taken to be about 60 or 70 km (e.g. [35,38,40]); calculations show that a certain variation of this value does not substantially affect the resulting distribution of the electric potential, as this potential does not change much with height at ionospheric heights.

In order to find the value of *j_z_*, charge generation regions need to be considered as well (i.e. regions occupied by thunderstorms and ESCs), since thunderstorm regions and fair-weather regions are linked together in the GEC and hence a complete set of equations for the electric potential *ϕ* in the atmosphere is global in its nature. This is because charge separation within thunderstorms and ESCs maintain the current structure in the whole atmosphere, and the value of the fair-weather current density *j_z_* is determined by the entire conductivity distribution in the atmosphere together with the distribution of thunderstorm generators. However, here the problem is only considered locally in the fair-weather region alone. Therefore, the value of *j_z_* is regarded as a quantity provided by the global circuit to the local region considered.

Because water droplets remove the air ions principally responsible for air's conductivity by attachment and, as already described, because heavier water droplets are much less mobile electrically than the lighter air ions, the in-cloud conductivity *σ*_c_ is much less than the conductivity of the clear air *σ*_0_. If the transition distance from clear air to cloudy air is *D*, the charge density can be approximated from (4.6) as [18]

4.9 $$|\rho |\approx \frac{\varepsilon_{0}|j_{z}|}{\sigma_{\text{c}}^{2}}\frac{\sigma_{0}-\sigma_{\text{c}}}{D}=\frac{\varepsilon_{0}|j_{z}|}{D\sigma_{0}}\frac{1-K}{K^{2}},$$

where *K* = *σ*_c_/*σ*_0_ (cf. [43]). Assuming, following Nicoll & Harrison [20], that *K* ∼ 0.2 and *D* ∼ 100 m, and inserting −2 pA m^−2^ and 40 fS m^−1^ as typical values of *j_z_* and *σ*_0_, respectively, this gives a rough estimate for |*ρ*| of about 90 pC m^−3^. This is broadly consistent with the measured space charge values shown in figure 1. In the absence of dynamical mixing within the cloud, this charge density will be negative at the layer cloud base and positive at the cloud top.

To analyse the same problem more consistently, we employ equation (4.6). It is particularly instructive to compare the situation where a layer cloud is present with that where there is no cloud. Considering first the cloud-free case, we suppose, for simplicity, that the undisturbed conductivity distribution in the atmosphere can be represented as

4.10 $$\sigma_{0}(z)=\sigma_{00}\;\exp\left(\frac{z}{H}\right)$$

with surface conductivity *σ*_00_ = 40 fS m^−1^ and scale height *H* = 6 km. This is a simple profile approximating more complicated conductivity representations used in GEC modelling; specific values of *σ*_00_ and *H* are those from Tinsley & Zhou [23]. (In particular, PBL effects are neglected, which are extremely variable over the Earth's surface.) Extrapolating conductivity profiles from [23, fig. 10] corresponding to the altitude range above approximately 5 km down to the Earth's surface gives *σ*_00_ ∼ 10 fS m^−1^. A more detailed analysis using equations from [23] shows that 40 fS m^−1^ provides a good estimate of the mean conductivity at the Earth's surface if effects of radon and aerosols, which vary substantially across different land and ocean locations, are neglected. For the characteristic scale height *H* for the conductivity, the profiles of [23, fig. 10] imply that this height is about 4 km below 15 km and then gradually increases beyond 10 km in the upper stratosphere; accordingly *H* = 6 km is assumed as an intermediate value for the lower atmosphere, where thunderstorm and ESC generators occur.

With the vertical current density being $j_{z}^{0}=-2$ pA m^−2^, and assuming *h*_u_ = 70 km, the profile (4.10) gives, according to (4.8), a value of *V*_u_ of about 300 kV, which is somewhat greater than the value of about 240 kV actually observed [30]. This is the result of the simple exponential conductivity profile (4.10) assumed instead of a more realistic but rather locally variable and complicated dependency, with *H* depending on *z*. High precision is not intended here as the actual value obtained does not affect the conclusions; to obtain a more realistic value of 240 kV, the magnitude of *j_z_* could be decreased by 20% or, alternatively, *σ*_00_ increased by the same amount.

In the other case, we suppose that the region between *h*_bot_ = 800 m and *h*_top_ = 1200 m is occupied by a cloud, using typical cloud parameters observed at Reading (see Nicoll & Harrison [20], and therefore the conductivity *σ*(*z*) in this region is reduced in accordance with the discussion above. This is expressed by assuming that

**Figure 5.**
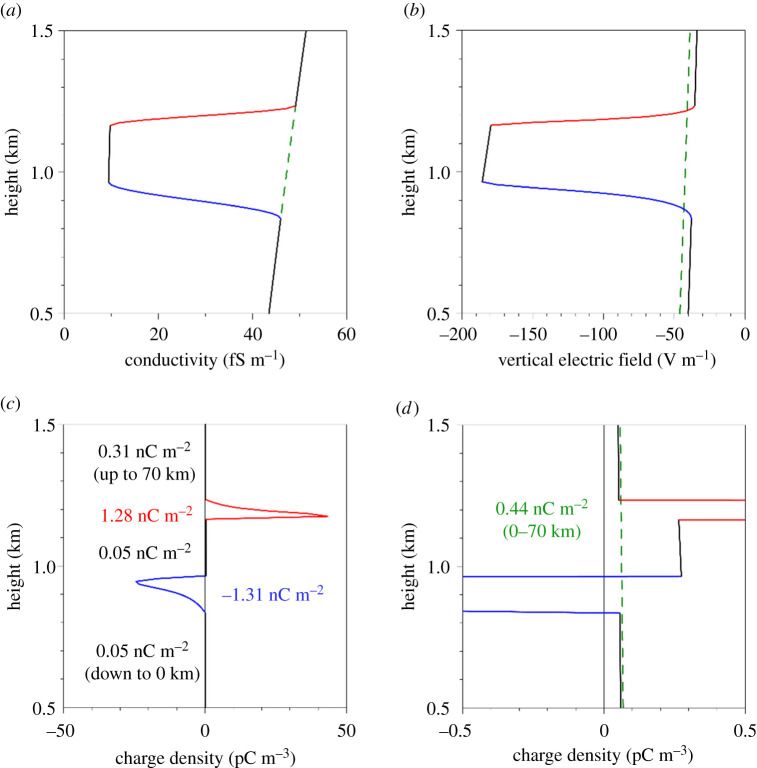
Estimated vertical profiles of atmospheric electrical quantities in the lower atmosphere with a thin, horizontally extensive layer cloud present at an altitude of 1 km: (*a*) assumed vertical profile of the conductivity, (*b*) corresponding vertical profiles of the electric field intensity and (*c*,*d*) calculated charge density ((*d*) is the close-up of (*c*)). Also shown are the total charges of the different regions per unit area. Red and blue sections of the plots indicate the positively and negatively charged transition regions between the interior of the cloud and the clear air. Green dashed lines correspond to the case where the cloud is absent (assuming the same value of the ionospheric potential). (Online version in colour.)

4.11 $$\sigma (z)=\sigma_{0}(z)\times \left\{ \begin{array}{ll} K, & h_{\text{bot}}<z<h_{\text{top}},\\ 1, & \text{otherwise} \end{array}\right.$$

with *K* = 0.2. Moreover, the transitions from *K* to 1 of the coefficient are smoothed in this formula, using appropriate pieces of sine curves, the transition distances being (see [20]) *D*_bot_ = 130 m at the lower boundary of the cloud (i.e. 65 m above and below *z* = *h*_bot_) and *D*_top_ = 70 m at its upper boundary (i.e. 35 m above and below *z* = *h*_top_). This smoothed conductivity profile is shown in figure 5*a* (red and blue sections indicate transition regions), in which the green dashed line shows the profile unperturbed by cloud. Note that this does not account for possible effects of the PBL on the conductivity profile: these are discussed further at the end of this section.

The introduction of a single cloud has a negligible effect on the ionospheric potential *V*_u_, which is determined by the global structure of distant thunderstorm and ESC generators in the entire atmosphere and by the global fair-weather resistance. However, given that the resistance of an air column containing cloud is greater than that of a cloud-free column, the vertical current must be smaller in the cloudy column than in the clear column. Inferring from (4.8) that

4.12 $$V_{\text{u}}=-j_{z}\int_{0}^{h_{\text{u}}}\frac{\text{d}z}{\sigma (z)}=-j_{z}^{0}\int_{0}^{h_{\text{u}}}\frac{\text{d}z}{\sigma_{0}(z)}$$

and calculating the integrals, we conclude that if in the absence of clouds $j_{z}^{0}=-2$ pA m^−2^ then in the presence of a cloud *j_z_* = −1.74 pA m^−2^ (for the same global supply current and the same *V*_u_).

For the case where a cloud is present, the vertical profiles of the electric field and charge density calculated by means of formulae (4.4) and (4.6) are shown in figure 5*b*,*c*, respectively. The latter figure also presents total charges per unit area of different regions (shown by alternating colours); in the calculation of the charge of the uppermost region it has been assumed that the upper boundary is set at *h*_u_ = 70 km, and in the calculation of the charge of the lowermost region it has been assumed that the lower boundary is set at 0 km. The green dashed line in figure 5*b* shows the electric field profile in the no-cloud case; however, it seems nearly impossible to show the corresponding charge density profile in figure 5*c* on the same axis scale. This is presented in figure 5*d*, where the behaviour of the two charge density profiles is compared outside the two transition regions; this figure also indicates the total charge per unit area in the no-cloud case.

Notwithstanding the fact that the absolute value of the charge density at the upper cloud boundary is greater than that at the lower boundary, the total charge of the upper transition region is clearly smaller than that of the lower transition region. Also, the total charge of the entire cloud (including its interior and two transition zones) is positive. This is not surprising, since, by integrating the equation

4.13 $$\rho (z)=\varepsilon_{0}j_{z}\frac{\text{d}}{\text{d}z}\left(\frac{1}{\sigma }\right)(z)=-\varepsilon_{0}|j_{z}|\frac{\text{d}}{\text{d}z}\left(\frac{1}{\sigma }\right)(z),$$

the charge per unit area of the region enclosed between *z* = *a* and *z* = *b* is given by

4.14 $$Q[a\leq z\leq b]=-\varepsilon_{0}|j_{z}|\int_{a}^{b}\frac{\text{d}}{\text{d}z}\left(\frac{1}{\sigma }\right)(z)\;\text{d}z=\varepsilon_{0}|j_{z}|\left(\frac{1}{\sigma (a)}-\frac{1}{\sigma (b)}\right).$$

As the conductivity above the cloud is evidently greater than the conductivity below the cloud, the total charge of the cloud is positive (in agreement with the data shown in figure 2*b*). Note that the charge of the region *a* ≤ *z* ≤ *b* is determined by *σ*(*a*) and *σ*(*b*) and does not depend on the conductivity profile in the segment *a* ≤ *z* ≤ *b* (in the absence of non-conductive currents, e.g. without allowing for the charge separation occurring inside electrified clouds).

Finally, the total charge per unit area of the column (from 0 km up to *h*_u_ = 70 km) equals

4.15 $$Q[0\leq z\leq h_{\text{u}}]=\varepsilon_{0}|j_{z}|\left(\frac{1}{\sigma (0)}-\frac{1}{\sigma (h_{\text{u}})}\right)=\frac{\varepsilon_{0}|j_{z}|}{\sigma_{00}}\left(1-\exp\left(-\frac{h_{\text{u}}}{H}\right)\right),$$

or 386.167 pC m^−2^. In comparison, the interface conditions for the electric field at the Earth's surface imply that its surface charge density

4.16 $$s=\varepsilon_{0}E_{z}(0)=\varepsilon_{0}\frac{j_{z}}{\sigma (0)}=-\frac{\varepsilon_{0}|j_{z}|}{\sigma_{00}},$$

or −386.170 pC m^−2^ (clearly *E_z_*(0) here is the same as *E*_s_ in equation (2.1)). Thus the two charges do not compensate for each other completely; the small difference between their absolute values,

4.17 $$-s-Q[0\leq z\leq h_{\text{u}}]=\frac{\varepsilon_{0}|j_{z}|}{\sigma_{00}}\exp\left(-\frac{h_{\text{u}}}{H}\right)=-\varepsilon_{0}\frac{j_{z}}{\sigma (h_{\text{u}})}=-\varepsilon_{0}E_{z}(h_{\text{u}}),$$

is equal to the surface charge density at the upper boundary of the atmosphere (set in our model at *h*_u_), provided we assume no electric field outside the model atmosphere.

By comparing the total charge in the air column in the presence of a cloud with that in the cloudless case (figure 5*c*,*d*) we observe that the former is smaller than the latter. This can be easily explained by noting that the charge of the air column containing a cloud is determined by equation (4.15), whereas in the no-cloud case it should be described by the same equation with |*j_z_*| being replaced by $|j_{z}^{0}|<|j_{z}|$.

**Figure 6.**
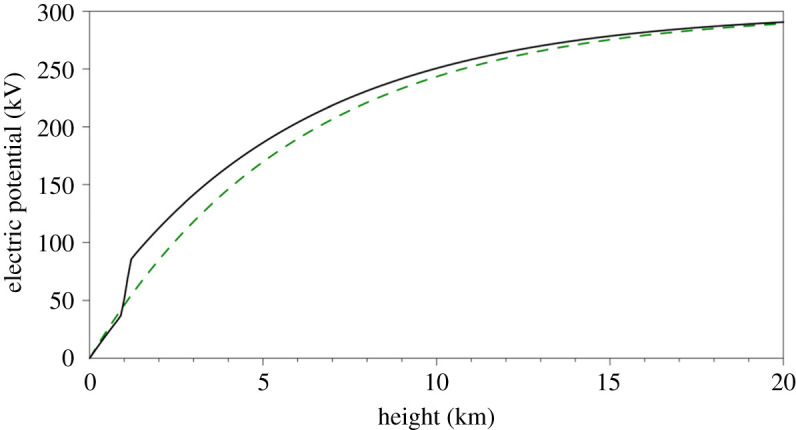
Estimated vertical profiles of the electric potential in the lower atmosphere, in the presence of a layer cloud (black solid line) and in the no-cloud case (green dashed line). (Online version in colour.)

Figure 6 compares the vertical profiles of the electric potential, estimated from equation (4.7) in the presence and in the absence of a cloud. This figure illustrates how two somewhat different potential profiles eventually reach the same value of *V*_u_. This occurs because the integrals of *E_z_* are the same, as enhanced electric field values inside the cloud are balanced by reduced values above and below the cloud. It is also evident that, above the layer cloud, a given potential is reached at a lower altitude than where it would be reached in the cloudless case. This may have some small practical benefit in reducing the height required for soundings intended to obtain *V*_u_, but only if the resistive regions are sufficiently homogeneous and horizontally extensive that the above-cloud and below-cloud regions encountered can accurately compensate for the changes in the charge distribution (e.g. [44]).

In the discussion above, the PBL was not considered, where the conductivity is often reduced. Although the PBL is a very important aspect for atmospheric electricity, its properties vary substantially across different land and oceanic locations [45]. From a theoretical viewpoint, the PBL influences the GEC not only via conductivity reduction but also by the fact that processes occurring within it may result in additional charging (non-conduction) currents serving as generators contributing to the GEC along with thunderstorms and ESCs [8,46].

As discussed in the Introduction, appropriate detailed study of all these issues lies beyond the scope of our present research, which is driven by the observations available; we note, however, that, for the particular cloud layer considered here, the PBL height is effectively at the top of the cloud layer. Since rapid charge density and conductivity changes in the vicinity of the cloud are not observed, other than those related to the cloud itself [20, fig. 2*c*,*d*], we conclude that the meteorological conditions are such that the charge in the PBL is well mixed. Thus, there is no distinct layer of space charge at the PBL top, as has been observed in clear air conditions by other investigators [47]. Hence, for this particular situation, the reduction of conductivity in the PBL with height is gradual rather than marked. It is likely that in the theoretical analysis performed in this section we have overestimated the conductivity in the vicinity of the Earth's surface and, by inference, underestimated the electric charge stored in this region (according to equation (4.14)) and the electric field magnitude at the Earth's surface (according to equation (4.4)); however, this does not affect our conclusions regarding the charge structure in the neighbourhood of such a cloud, which is the purpose of this study.

## 5. Conclusion

Stratiform layer clouds cover a substantial proportion of the planet at any given time and influence their local electrical environment, yet their presence is typically neglected in the conventional conceptual pictures of the GEC. The ability of layer clouds to perturb the vertical profiles of conductivity, space charge density and potential compared with those of the fair-weather atmosphere has motivated us to make a closer examination of the traditional conceptual picture of how the charge is distributed within the GEC. A layer cloud acquires positive charge at its upper boundary and negative charge at its lower boundary and contains positive charge in its interior. Hence the meteorological processes leading to layer cloud formation also yield consequences for the atmospheric charge structure. Note that the PBL is another very important aspect of ‘semi-fair-weather’ electricity that can also affect charge balance in the atmosphere, but PBL properties vary so substantially across different locations that a full investigation is beyond the scope of this article.

The total column charges accumulated at the layer cloud boundaries differ, with the upper boundary column charge being the greater in magnitude, but they are of the same order and reach several nC m^−2^. Taking the absolute value of the median cloud base surface charge density of −1 nC m^−2^ (figure 4) from Reading as an approximation for the cloud top charge (1 nC m^−2^), it is possible to estimate the total charge present worldwide on the upper boundaries of stratiform clouds. If 30% of the planet is covered by such clouds [24], the total positive charge stored by these clouds at their upper boundary would be 1.5 × 10^5^ C.

The total charge accumulated within the cloud (when its interior is considered together with the two transition regions) is always positive, and may reach tens to hundreds of pC m^−2^. Theoretical calculations also show that the total (positive) charge of a fair-weather column, even if layer cloud is present, is almost precisely compensated by the negative charge accumulated at the Earth's surface below this column. Although these findings have been obtained under the assumption that the undisturbed conductivity profile can be parameterized by an exponential form (equation (4.10)) with a surface conductivity 40 fS m^−1^ and scale height 6 km, our ultimate conclusions regarding charge balance in the fair-weather column do not rely on their specific values (or even on the specific equation describing the conductivity profile).

**Figure 7.**
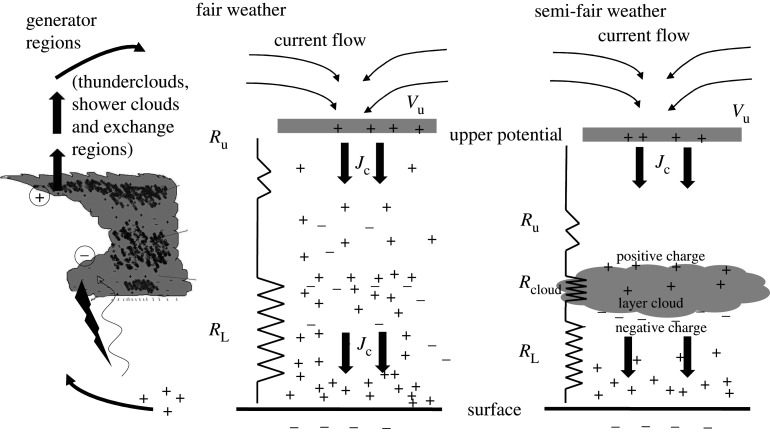
Description of the current flow in the global electric circuit in the fair-weather and semi-fair-weather regions. *Left panel*: charge separation in thunderclouds, shower clouds and other exchange regions in disturbed weather regions drives upward current between the surface and the upper atmosphere (lightning is shown from the cloud base, the wavy arrow indicates point discharge currents, i.e. corona); *middle panel*: downward return current flow *J*_c_ in a cloudless fair-weather region showing the upper potential *V*_u_ and the effective resistance of the lower atmosphere *R*_L_ (comprising the PBL) and upper troposphere *R*_u_ (i.e. above the cloud-forming regions); *right panel*: downward return current flow in a region containing layer cloud (with resistance *R*_cloud_) through which the return current passes. In the semi-fair-weather region, the upper potential is reached at a slightly lower altitude than in the cloudless case.

Extensive layer clouds are, in both abundance and function, an intrinsic part of the fair-weather branch of the GEC. Figure 7 depicts a new representation of current flow in the GEC which takes account of both fair-weather and semi-fair-weather regions. The middle and right panels in figure 7 represent the return current flow (generated in the disturbed weather regions—left panel in figure 7) in the fair-weather and semi-fair-weather regions, respectively. Note that in each situation the positive space charge is regarded as being distributed throughout the column, rather than being solely located in the base of the electrosphere. In the case of the semi-fair-weather atmosphere, in which an extensive cloud layer is distinct, most of the charge is located beneath the cloud top, leading to the upper potential being reached at a lower altitude than in the cloudless case. The effect of layer clouds is to draw charge from the global circuit onto cloud droplets in an ordered way. For the smallest droplets at least, their behaviour, which may be relevant to the layer clouds' effects on climate, can be strongly influenced by electric forces.

## Footnotes

1 The widespread use of the term ‘global circuit’ began at the end of the 1960s. Before then, the global atmospheric electrical system was variously described as the ‘equivalent circuit', or ‘classical picture', sometimes summarized quantitatively by an ‘electrical balance sheet'.

## Data accessibility

The balloon flight data used in the analysis are archived through the University of Reading Data Repository at http://dx.doi.org/10.17864/1947.38. Reading surface electric field data are available through the GloCAEM database (NERC project grant no. NE/N013689/) at http://data.ceda.ac.uk//badc/glocaem/data/ and cloud base data through https://metdata.reading.ac.uk/ext/data_extractor.
